# Specific lipofuscin staining as a novel biomarker to detect replicative and stress-induced senescence. A method applicable in cryo-preserved and archival tissues

**DOI:** 10.18632/aging.100527

**Published:** 2012-12-29

**Authors:** EA Georgakopoulou, K Tsimaratou, K Evangelou, Marcos-PJ Fernandez, V Zoumpourlis, IP Trougakos, D Kletsas, J Bartek, M Serrano, VG Gorgoulis

**Affiliations:** ^1^ Molecular Carcinogenesis Group, Department of Histology and Embryology, School of Medicine, University of Athens, Greece; ^2^ Tumor Suppression Group, Molecular Oncology Program, Spanish National Cancer Centre (CNIO), Melchor Fernández Almagro 3, Madrid E-28029, Spain; ^3^ National Hellenic Research Foundation, Institute of Biological Research and Biotechnology, Athens, Greece; ^4^ Department of Cell Biology and Biophysics, Faculty of Biology, University of Athens, Panepistimiopolis, Athens 15784, Greece; ^5^ Laboratory of Cell Proliferation and Ageing, Institute of Biology, National Centre for Scientific Research ‘Demokritos’, Athens, Greece; ^6^ Danish Cancer Society Research Center, Copenhagen, Denmark; ^7^ Institute of Molecular and Translational Medicine, Faculty of Medicine and Dentistry, Palacky University, Olomouc, Czech Republic; ^8^ Biomedical Research Foundation, Academy of Athens, 11527 Athens, Greece

**Keywords:** lipofuscin, cellular senescence, Sudan Black B, formalin-fixed paraffin-embedded, biomarker

## Abstract

There is shortage of extensive clinicopathologic studies of cellular senescence because the most reliable senescence biomarker, the detection of Senescence-Associated-beta-galactosidase activity (SA-β-gal), is inapplicable in archival material and requires snap-frozen tissues. We validated the histochemical Sudan-Black-B (SBB) specific stain of lipofuscin, an aggregate of oxidized proteins, lipids and metals, known to accumulate in aged tissues, as an additional reliable approach to detect senescent cells independently of sample preparation. We analyzed cellular systems in which senescence was triggered by replicative exhaustion or stressful stimuli, conditional knock-in mice producing precancerous lesions exhibiting senescence, and human preneoplastic lesions known to contain senescent cells. In the above settings we demonstrated co-localization of lipofuscin and SA-β-gal in senescent cells *in vitro* and *in vivo* (cryo-preserved tissue), strongly supporting the candidacy of lipofuscin for a biomarker of cellular senescence. Furthermore, cryo-preserved tissues positive for SA-β-gal were formalin-fixed, paraffin-embedded, and stained with SBB. The corresponding SA-β-gal positive tissue areas stained specifically for lipofuscin by SBB, whereas tissues negative for SA-β-gal were lipofuscin negative, validating the sensitivity and specificity of the SBB staining to visualize senescent cells in archival material. The latter unique property of SBB could be exploited in research on widely available retrospective tissue material.

## INTRODUCTION

Cellular senescence is the state of irreversible cellular growth arrest in which the cell remains metabolically active [1, 2]. Two types of cellular senescence have been described in mammalian cells [3]. Replicative Senescence (RS) that is triggered by the arrest of cellular proliferation after a certain number of divisions due to telomere attrition [1, 3] and Stress Induced Premature Senescence (SIPS) that is a more acute phenomenon in which the cells stop to proliferate under various stress conditions, regardless of telomere length [3]. The phenomenon of cellular senescence was originally described *in vitro* [4]. More recently, senescent cells were also identified in aged skin [2], benign tumors and premalignant lesions [5-9] as well as in age-related pathologies [10]. Also, the number of senescent fibroblasts reportedly increases exponentially in the skin of aging primates, reaching >15% of all cells in very old individuals [11]. The evidence so far from *in vitro* and *in vivo* studies suggests that cellular senescence acts as a tumor barrier, whereas it contributes to the processes of tissue aging and age-related diseases [12]. The significance of cellular senescence incarcinogenesis and age-related disorders, renders the detection of these phenomena essential. This urgent need of reliable biomarkers of senescence is even more apparent given the evidence for cellular senescence induced in response to anticancer therapy [13].

The most widely used biomarker of cellular senescence reported so far is the detection of Senescence-Associatedβ-Galactosidase activity (SA-β-gal) in sub-optimal pH [2, 14]. Nevertheless, a major disadvantage in designinglarge-scale studies of cellular senescence in humanlesionsis that SA-β-gal staining requires fresh tissue as it is based on an enzymatic reaction [14]. This fact seriously limits the exploitation of the widely available formalin-fixed paraffin-embedded (FFPE) archival tissues, including tissue microarrays [1]. In an effort to establish a biomarker of cellular senescence that could be applicable for FFPE archival tissue material, we focused on lipofuscin, also known as an "age-pigment" [15]. Lipofuscin is an aggregate of oxidized proteins that accumulates progressively mostly in aged post mitotic cells [16]. It is considered a hallmark of aging and is also involved in the pathogenesis of certain age related pathologies such as macular degeneration [16]. Sudan Black B (SBB) is a lipophilic histochemical stain that identifies lipofuscin and is applicable for *in vitro* and *in situ* studies [17-19]. Here we employed SBB in a series of experiments designed to demonstrate that lipofuscin accumulates *in vitro* in normal human cells during RS or SIPS, as well as in stressed human cancer cells. Furthermore, we sought to identify lipofuscin deposits in benign lesions already known to contain senescent cells. As a control marker of the cellular senescence state we used the SA-β-gal assay. Our results show that the SBB-stained lipofuscin is present in all the cells that express SA-β-gal activity and it is absent in SA-β-gal-negative cells. Hence, SBB positivity could be used as an additional cellular senescence biomarker. Moreover, SBB staining was applicable in FFPE tissue sections, providing evidence that this assay can provide a reliable biomarker for detection of senescent cells in archival clinical material that is stored in paraffin.

## RESULTS

**Figure 1:**
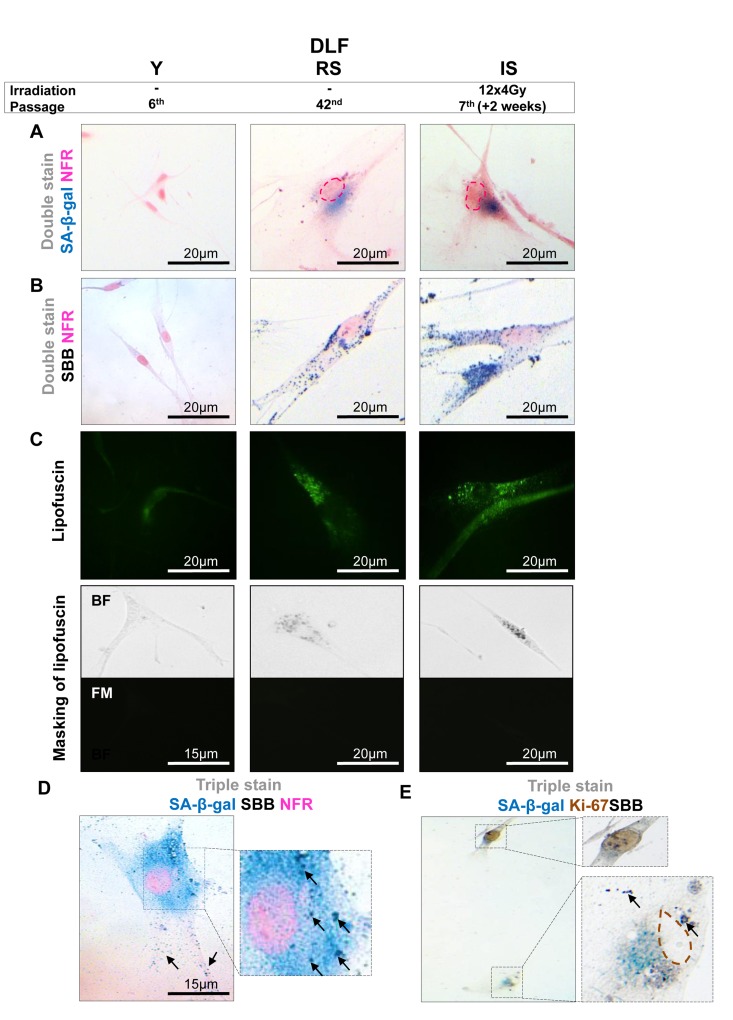
Lipofuscin accumulates and co-localizes with Senescence-Associated beta-galactosidase (SA-β-gal) in sub-confluent senescent primary human diploid lung fibroblasts (DLF) **Y** (Young): Early- passage cells, **RS:** Replicative-senescent cells and **IS** (irradiated): Early passage cells that became prematurely senescent after irradiation (12×4Gy). Collected cells were fixed on slides with 4% parafolmadehyde (**A**) All three cultures were stained with SA-β-gal and nuclear fast red as counterstain (NFR). Cells from RS and IS cultures acquired the characteristic senescent morphological phenotype (enlarged and flattened) and were positive for SA-β-gal staining (turquoise color). (**B**) All cultures were stained with Sudan Black B (SBB) and NFR. Cells from RS and IS cultures, which had the morphological phenotype of senescence, were also positive for SBB (dark blue-black granules). (**C**) **Top panels:** green pseudocolor represents visualization of lipofuscin's autofluorescence at 450-490 nm. **Bottom panels:** RS and IS cells that stained with SBB (BF, bright field microscopy) show no auto-fluorescence of lipofuscin (FM, fluorescence microscopy without pseudocolor), indicating that SBB stains lipofuscin. Cells with the morphological phenotype of senescence were positive for both SA-β-gal and SBB (**D**), while cells that were positive for Ki67 were negative for both SA-β-gal and SBB (**E**). Insets: Cells at higher magnification, pink dashed lines: indicate NFR-stained nuclei, brown dashed lines: indicate Ki67- negative nuclei, black arrows: show SBB granules.

**Figure 2:**
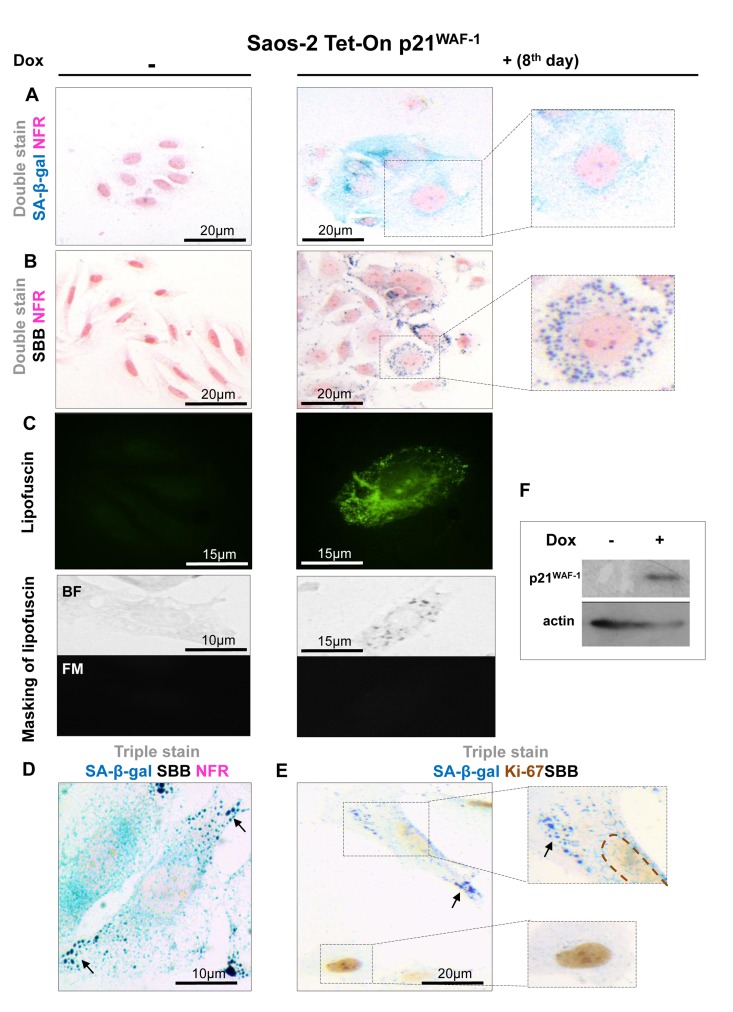
Lipofuscin accumulates and co-localizes with Senescence-Associated beta-galactosidase (SA-β-gal) in senescent Saos-2 cells triggered by p21^WAF-1^ (**A**) SA-β-gal staining (turquoise color) in the Saos-2 p21^WAF-1^ Tet-On cell system on the 8^th^ day of doxycycline (5 μg/ml) addition. **Inset**: Senescent cells acquired the characteristic senescent morphological phenotype (enlarged and flattened). (**B**) Sudan Black B (SBB) positivity (dark blue-black granules) in cells with senescent morphological phenotype (**inset**). (**C**) **Top panels**: Lipofuscin's auto-fluorescence in induced Saos-2 p21^WAF-1^ Tet-On cells, by fluorescence microscopy at 450-490 nm (green pseudocolor). **Bottom panels**: Cytochemical SBB staining (BF, bright field microscopy) quenches the auto-fluorescence of lipofuscin (FM, fluorescence microscopy), indicating that SBB stains lipofuscin. SA-β-gal and SBB staining coincided in cells that had the morphological phenotype of senescence (**D**) and were absent in cells that were positive for the proliferative marker Ki67 (**E**). (**F**) Addition of doxycyclin (Dox) triggers p21^WAF-1^ expression. Brown dashed lines: Ki67- negative nuclei. Black arrows: SBB granules. NFR: nuclear fast red counterstain.

To assess the value of lipofuscin as a potential biomarker of cellular senescence *in vitro*, five cellular systems of normal diploid cells and cancer cells were applied. In these experimental settings cellular senescence was triggered by means of proliferative exhaustion (Replicative Senescence, RS) or Stress Induced Premature Senescence (SIPS). Specifically, we used young proliferating primary human diploid lung fibroblasts (DLF) (at passage 6), along with replicatively senescent cells (at passage 42) and cells (at passage 7) in which SIPS was triggered by γ-irradiation [20] (Fig. 1). The effect of p53 and p21^WAF-1^, two well established effectors of the senescence program, was studied in two inducible osteosarcoma cell lines, namely Saos-2 p21-Tet-ON and Saos-2 p53-Tet-ON [21-24]. After 8 days of p21^WAF-1^ or p53 induction senescent cells were evident (Fig. 2A, 3A). Finally, the inducible osteosarcoma U2OS E2F1-ER cell line was also tested. Over-expression of E2F1 has been shown to trigger SIPS via activation of the DNA damage response (DDR) pathway [25]. The induction of E2F1 yielded senescent cells in 10 days (Fig. 4A) as concluded by positive SA-β-gal staining.

**Figure 3:**
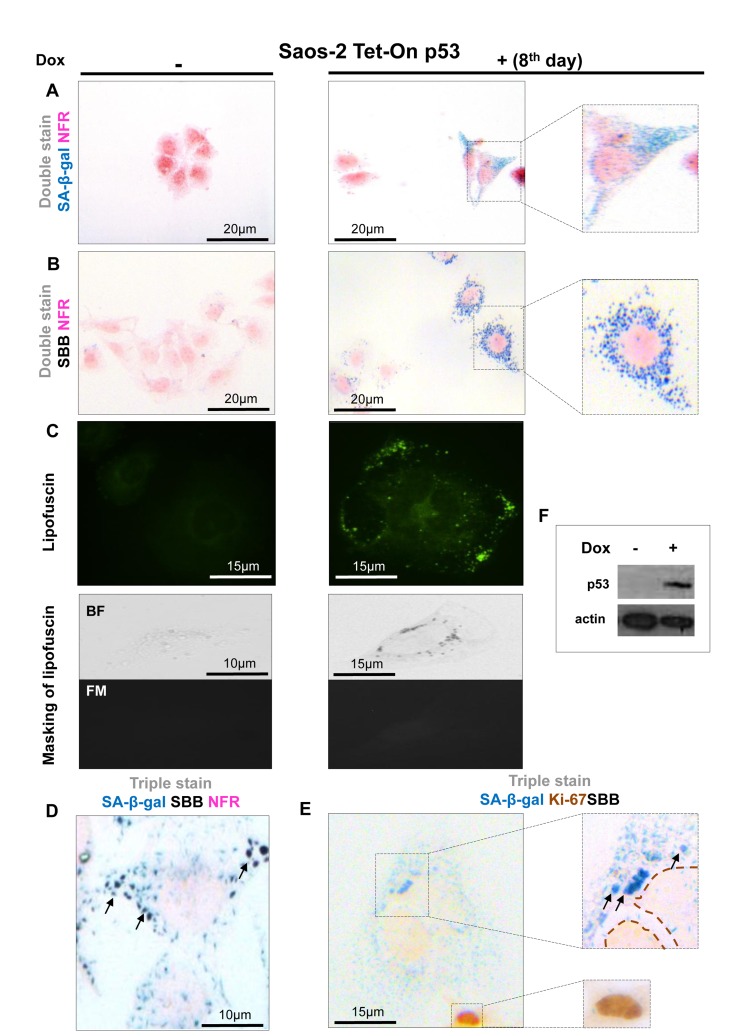
Lipofuscin accumulates and co-localizes with Senescence-Associated beta-galactosidase (SA-β-gal) in p53-mediated Saos-2 senescent cells (**A**) Senescent cells with the characteristic morphology (enlarged and flattened) and positivity for SA-β-gal staining (turquoise color), on the 8^th^ day of induced with doxycycline of the Saos-2 p53 Tet-On system. (**B**) Sudan Black B (SBB) perinuclear accumulation as dark blue-black granules, in cells with senescent morphology. (**C**) **Top panels**: Perinuclear appearance of lipofuscin in apparently senescent cells: pseudocolor visualization of lipofuscin's auto-fluorescence (450-490 nm) is represented in green. **Bottom panels**: Lipofuscin's auto-fluorescence (FM, fluorescence microscopy) is masked by SBB staining (BF, bright field microscopy). (**D**) Co-localization of SA-β-gal and SBB staining in senescent cells and, (**E**) Ki67 positive cells are negative for SBB and SA-β-gal. (**F**) Addition of 5μg/ml doxycyclin (Dox) leads to p53 expression. Brown dashed lines: Ki67- negative nuclei, black arrows: SBB granules, NFR: nuclear fast red counterstain.

**Figure 4:**
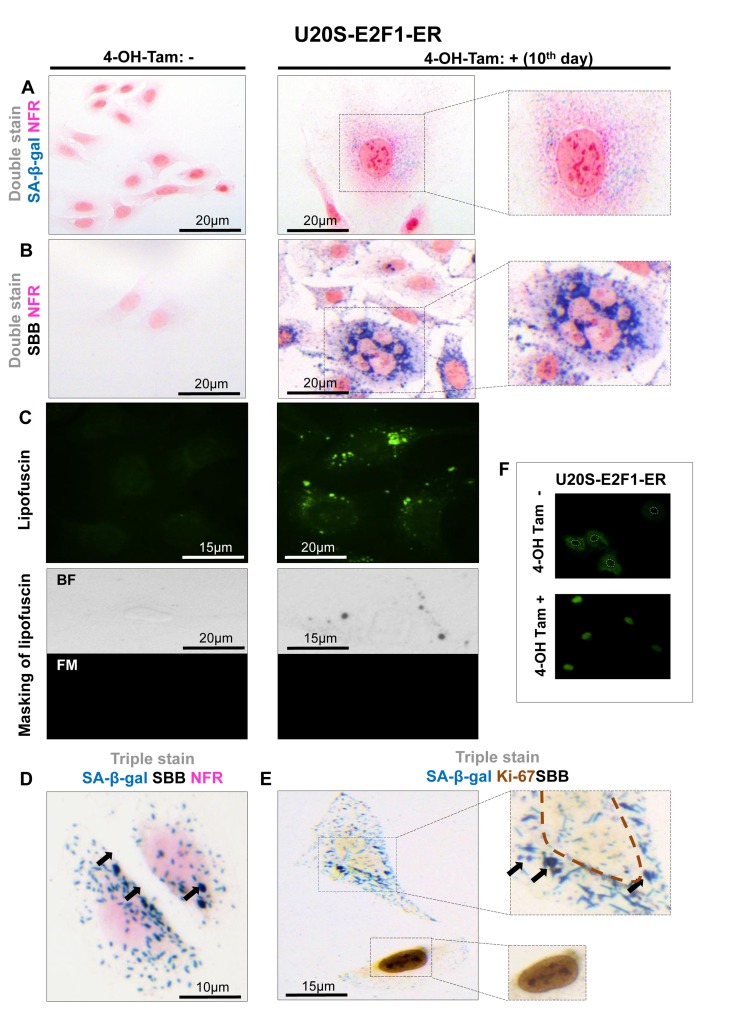
Lipofuscin accumulates and co-localizes with Senescence-Associated beta-galactosidase (SA-β-gal) in E2F-1 induced U2OS senescent cells (**A**) On the 10^th^ day of induction with 4-OH-Tamoxifen, cells were positive for SA-β-gal activity (turquoise color); cells also demonstrated the morphological phenotype of senescence (enlarged and flattened) (**B**) Cells demonstrating the characteristic senescent phenotype show Sudan Black B (SBB) dark blue-black granules (**C**) **Top panels**: Lipofuscin's auto-fluorescence at 450-490 nm is represented in green pseudocolor. **Bottom panels**: blocking of lipofuscin auto-fluorescence (FM, fluorescence microscopy) with SBB staining (BF, bright field microscopy) indicates that SBB stains lipofuscin (**D**) Concurrent positivity for SA-β-gal activity and SBB staining in the same cell, which is also negative for the proliferative marker Ki67 (**E**). (**F**) Addition of 300 nmol/L 4-OH-Tamoxifen (4-OH-Tam) leads to nuclear translocation of E2F1 (indirect immunofluorescence). E2F1-negative nuclei are indicated with white dashed lines. Brown dashed lines: Ki67- negative nuclei, black arrows: SBB granules, NFR: nuclear fast red counterstain.

In all the above systems cells that acquired the morphological features of senescence (i.e. became enlarged and flattened) were positive for SA-β-gal (Fig. 1A, 2A, 3A, 4A). These preparations were also stained with SBB and as illustrated in Fig. 1B, 2B, 3B and 4B all contained clearly visible perinuclear and cytoplasmic aggregates of lipofuscin, stained by SBB as dark blue-black granules. In addition, as lipofuscin produces auto-fluorescence, we could verify that the blue-black granules, stained by SBB, represented lipofuscin aggregates, by masking the lipofuscin's auto-fluorescence with SBB staining (Fig. 1C, 2C, 3C, 4C) [26]. This unique property of theSBB wasthe reason it was selected amongother methodsthat detectlipofuscin [19, 27]. To assess the extent of agreement between the results from the two assays, we performed SA-β-gal and SBB co-staining in the same cells. Indeed, co-staining results showed a complete overlap (Fig. 1D, 2D, 3D, 4D). Likewise, concomitant staining of SA-β-gal, SBB and the proliferative marker Ki67 verified that cells positive for lipofuscin and SA-β-gal activity were not proliferating (Fig. 1E, 2E, 3E, 4E).

**Figure 5:**
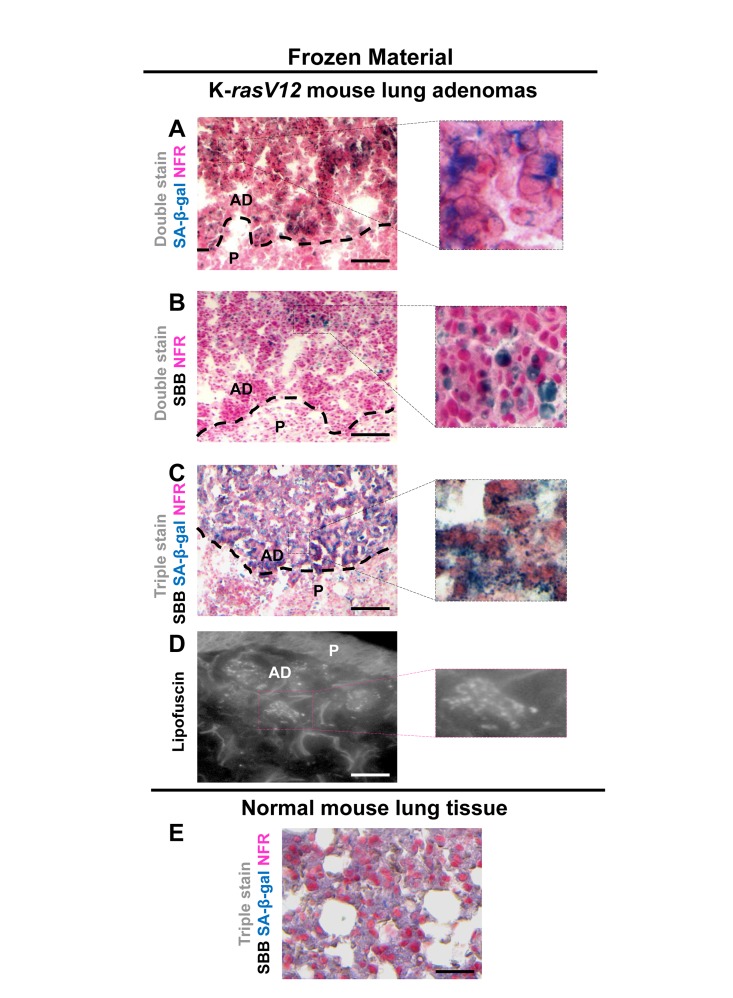
Lipofuscin and Senescence-Associated beta-galactosidase (SA-β-gal) activity co-localize in lung adenomas demonstrating senescence in a mouse model conditionally expressing K-*rasV12* in the lung Frozen sections derived from mouse lung K-rasV12 adenomas. (**A**) Cells from the adenomas show SA-β-gal activity. (**B**) Characteristic perinuclear deposition of blue black granules in cells stained with Sudan Black B (SBB), representing positivity for lipofuscin. (**C**) Cells from lung adenomas positive for both, SA-β-gal activity and lipofuscin. (**D**) Fluorescence microscopy (at 450-490 nm) verifying lipofuscin presence. (**E**) Normal mouse lung tissue negative for SA-β-gal activity and lipofuscin. **P**: Parenchyma, **AD**: Adenoma. Scale bars: A-C, 200 μm; D, 25 μm; E, 50 μm. Insets: Cells at higher magnification.

**Figure 6:**
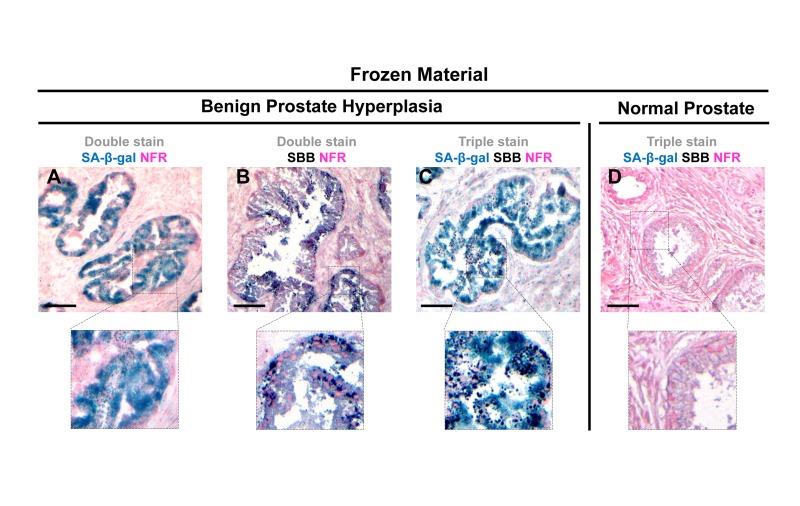
Lipofuscin accumulates and co-localizes with Senescence-Associated beta-galactosidase (SA-β-gal) in senescent cells detected in cryo-preserved material from benign prostatic hyperplasia (BPH) Frozen material from patients with BPH in enlarged prostates (prostate weight greater than 55gr) was thin-sectioned (5 μm). The sections were immediately double stained for SA-β-gal activity (turquoise color) and Nuclear Fast Red (NFR) as counterstain (**A**) and double stained with SBB and NFR (**B**). Areas with characteristic BPH pathology showed SA-β-gal activity and lipofuscin positivity (**C**). Normal prostate regions adjacent to BPH, were found negative for SA-β-gal activity and lipofuscin (**D**). Scale bars: A-C, 100 μm; D, 50 μm. Insets: Cells at higher magnification.

Next we examined whether SBB staining of lipofuscin could serve as a reliable senescence bio-marker *in vivo.* To this end the same experimental procedure was followed in frozen tissue sections from mice lung adenomas. The lung adenomas were developed in a mouse model expressing conditionally K-*rasV12*in the lung [5], one of the first *in vivo* settings used to demonstrate the role of senescence as an anti-tumor barrier in premalignant lesions [5]. In line with the *in vitro* findings, the lung adenomas that demonstrated strong SA-β-gal activity stained positive for lipofuscin while normal lung tissues were negative (Fig. 5). Next, we examined frozen human samples from patients with benign prostatic hyperplasia (BPH) from enlarged prostates (>55gr). These lesions had been previously shown to feature senescence [7, 28]. As shown in Fig.6, SA-β-gal activity and SBB staining co-localized, whereas adjacent normal prostatic glands were negative.

**Figure 7:**
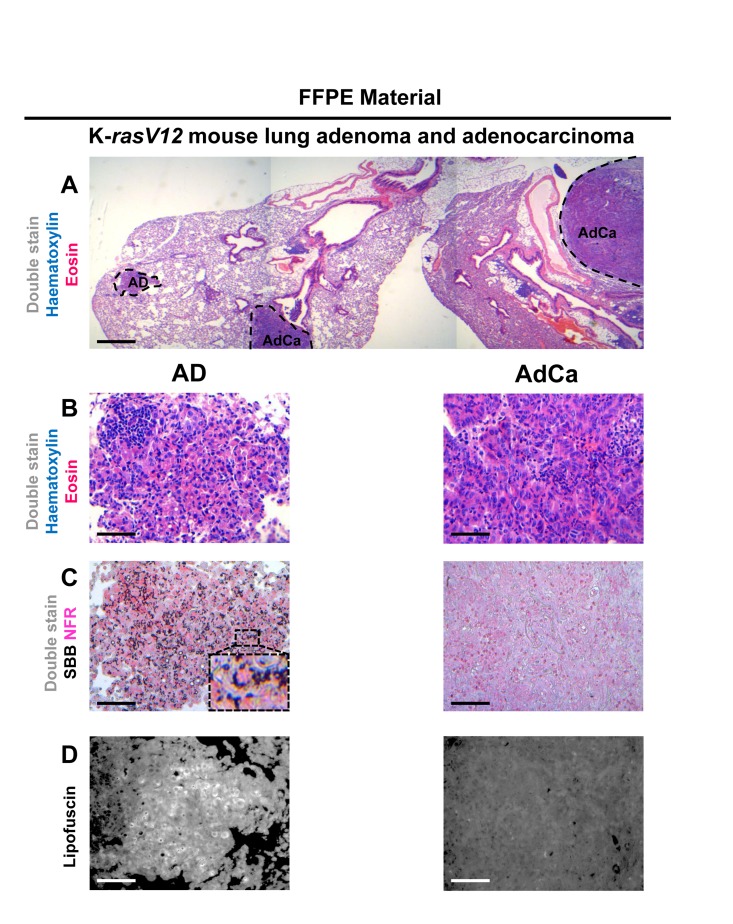
Sudan Black B (SBB) staining demonstrates lipofuscin accumulation in lung adenomas (AD) and absence in adenocarcinomas (AdCa), in formalin-fixed paraffin-embedded (FFPE) lung sections from mice conditionally expressing K-*rasV12* (**A**) Haematoxylin and Eosin staining demonstrates a lung adeoma (AD) and an adenocarcinoma (AdCa) on the same FFPE section (**B**) Histological features of the adenoma and the adenocarcinoma shown in **A** section. (**C**) Characteristic perinuclear deposition of blue black granules in adenoma cells stained with Sudan Black B (SBB), representing positivity for lipofuscin, while adenocarcinoma cells are negative for SBB. (**D**) Fluorescence microscopy (at 450-490 nm) verifying lipofuscin presence in the adenoma, and absence in the adenocarcinoma. Scale bars: A, 600 μm; B-D, 100 μm. Inset: Cells at higher magnification.

**Figure 8:**
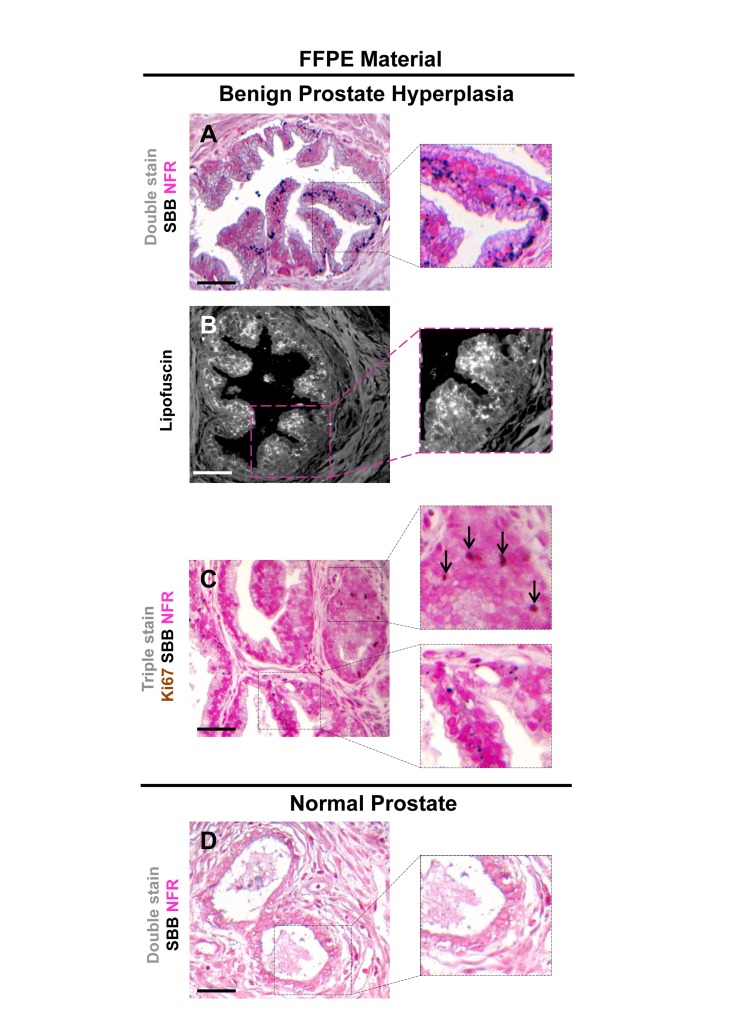
Accumulation of lipofuscin in formalin-fixed paraffin-embedded (FFPE) tissues from benign prostatic hyperplasia (BPH) that corresponds to senescent areas as depicted by Senescence-Associated beta-galactosidase (SA-β-gal) in cryo-preserved material FFPE sections from patients with BPH in enlarged prostates (prostate weight greater than 55gr) demonstrate accumulation of lipofuscin. Sections were deparaffinized and double stained with SBB (dark blue-black granules) and NFR as counterstain (**A**). Lipofuscin's presence was verified with fluorescence microscopy (**B**). Immunostaining for Ki-67 shows no matching with lipofuscin accumulation (**C**). Normal prostate regions adjacent to BPH, were negative for lipofuscin (**D**). Scale bars: BPH, 100 μm; Normal Prostate, 50 μm. Insets: Cells at higher magnification.

Having verified specific staining of senescent cells by SBB we then asked whether this approach could be also applicable in archival tissues. Thus, SBB stain was performed in FFPE tissue samples prepared from the above *in vivo* settings. As demonstrated in Fig.7, SBB staining clearly demonstrated lipofuscin in the lung adenomas (Fig.7). Strikingly, adjacent adenocarcinomas that spontaneously developed in these mice [5] were negative (Fig. 7). This finding supports the reliability of SBB as a method for staining senescent cells in FFPE (Fig. 7). Also, it is in line with the finding of Collado *et al.*, that spontaneously developing adenocarninomas in this model bypass the senescence anti-tumor barrier [5]. In addition, as representatively shown in Fig.8, SBB specifically stained blue-black lipofuscin granules in the FFPE material from BPH (see Fig.6) that were characterized as SA-β-gal positive. To further validate SA-β-gal and SBB staining tissue co-localization, we applied triple staining for SA-β-gal, SBB and NFR on BPH samples.

**Figure 9:**
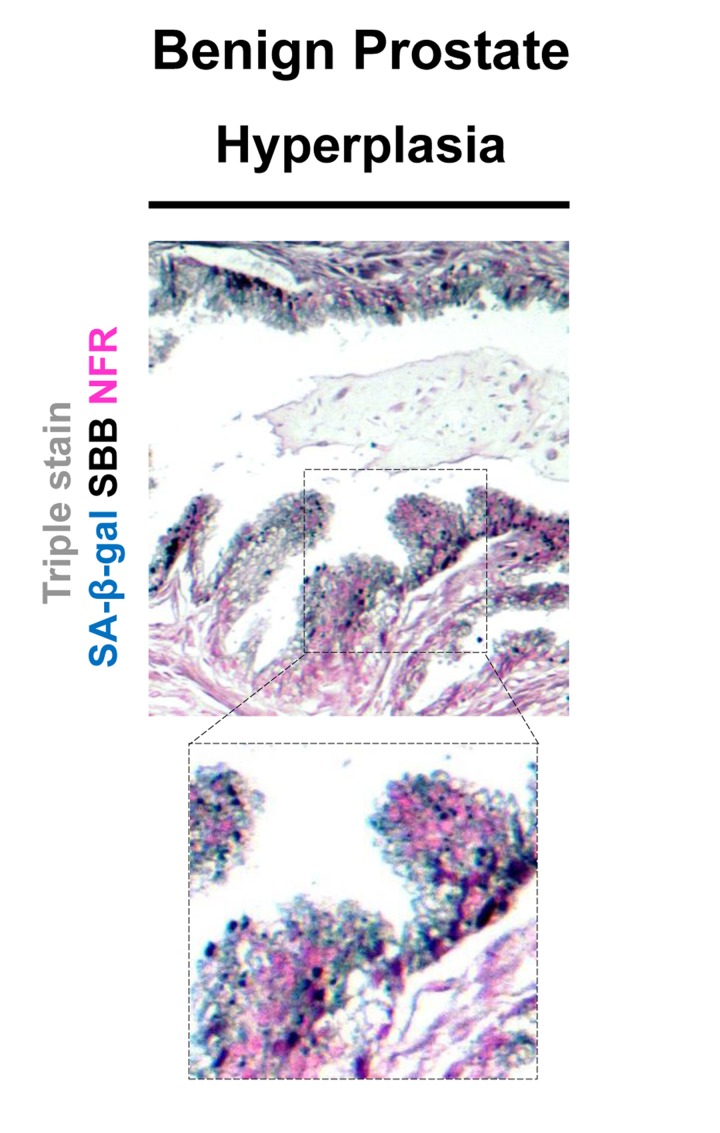
Co-localization of Senescence-Associated beta-galactosidase (SA-β-gal) activity and lipofuscin depiction in fresh-frozen tissue sample of benign prostatic hyperplasia (BPH) pretreated with SA-β-gal and subsequently embedded in paraffin Fresh samples with BPH were snap frozen, fixed in 4% formaldehyde, washed with buffer, incubated in SA-β-gal solution (turquoise color), subsequently fixed with formal-dehyde, and then embedded in paraffin, as previously shown (Michaloglou *et al* 2005). Sections where then double stained with Sudan Black B (SBB) (dark blue-black granules) and Nuclear Fast Red (NFR) as counterstain. Areas with the characteristic pathology of BPH showed SA-β-gal activity and lipofuscin positivity. Note the weak intensity of the Sa-β-gal staining.

Specifically, the frozen samples were fixed in formaldehyde for 2h, stained with SA-β-gal; routinely processed for FFPE as previously described [6], sectioned and then stained with SBB (Fig.9). Although,SA-β-gal activity was detected mostly in the periphery of the sections, leaving the core of the tissue unstained (likely due to slow penetration of the SA-β-gal stain into the tissue) a clear co-localization of SA-β-gal activity and SBB staining was noted close to the tissue periphery marked by pathological features of BPH. Interestingly, SBB foci were also observed in the core of the tissue (Fig. 9).

**Figure 10:**
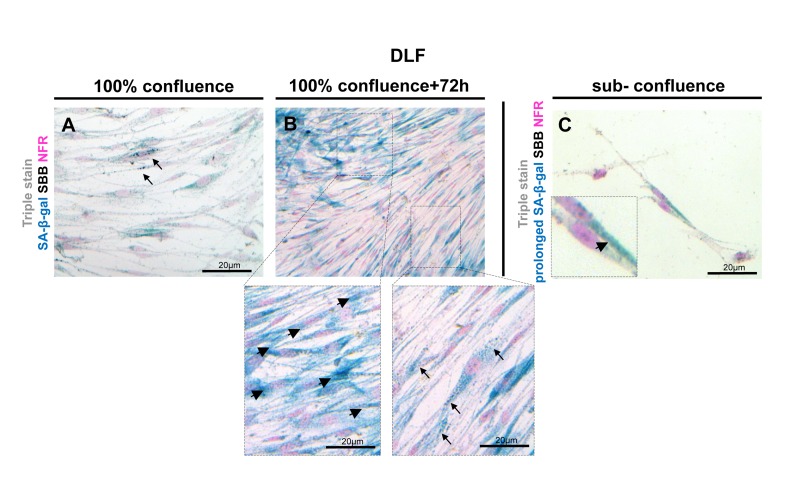
Lipofuscin staining and Senescence-Associated beta-galactosidase (SA-β-gal) activity in primary human diploid lung fibroblasts (DLF) Triple staining of early passage (6th) DLF cells with Sudan Black B (SBB) (dark blue-black granules), SA-β-gal (turquoise color) and Nuclear Fast Red (NFR), as counterstain. (**A**) Cells that had just reached 100% confluence showed SA-β-gal activity with negligible lipofuscin (arrows). (**B**) In the same assay 72 hours later, some cells demonstrated SA-β-gal staining (arrowheads) without lipofuscin, and there were also cells with concurrent SA-β-gal and SBB staining (arrows). (**C**) In sub-confluent DLF cells, prolonged SA-β-gal incubation (72 hours) followed by immediate SBB staining, demonstrated SA-β-gal activity (inset) without lipofuscin appearance.

It has been reported that false positive SA-β-gal staining may be detected in confluent cultures of quiescent cells [29, 30]. To investigate whether lipofuscin may be present in confluent cultures, we performed the SA-β-gal assay and SBB stain in confluent DLF cultures. We observed that as soon as the cells reached confluence they showed SA-β-gal activity while, in contrast, such cultures showed only negligible lipofuscin staining (Fig.10A). Extending the two assays for 72 hours we observed that all the cells clearly demonstrated SA-β- gal activity, whereas cells containing lipofuscin granules were fewer (Fig. 10B). Furthermore, the enzymatic activity of galactosidase [31] may produce positive results of SA-β-gal assay in non-senescent cells, when incubated for prolonged time. We incubated sub-confluent cultures of young DLFs in SA-β-gal for 72 hours (prolonged incubation) and then stained immediately with SBB. In these cells we observed SA-β -gal activity while there was no detectable SBB staining for lipofuscin (Fig. 10C).

## DISCUSSION

In this study, we examined whether lipofuscin, “a hallmark of aging”, [15] is also a cellular senescence biomarker and especially whether it is a marker of SIPS. Our working hypothesis was based on the notion that senescent cells accumulate in aged tissues [2]. We argued that the presence of lipofuscin in senescent cells could complement SA-β-gal by providing and additional marker of cellular senescence. To this end a series of experiments were designed in which various cellular systems, based on normal and cancer cells, were driven to either RS or SIPS. In line with our hypothesis all cells that displayed the senescence phenotype under either RS, or SIPS scenarios demonstrated co-localization of SA-β-gal activity and SBB-detected lipofuscin granules, both *in vitro* and *in vivo,* (Fig. 1D, 2D, 3D, 4D, Fig. 1E, 2E, 3E, 4E and Fig. 5C, 6C) whereas tissues negative for SA-β-gal activity were negative for lipofuscin, as well (Fig. 5E, 6D).

Lipofuscin is a non degradable aggregate of oxidized proteins, lipids and metals which accumulates inside the lysosomes of cells that do not replicate [16]. Such accumulation possibly reflects the inability of nonproliferating cells to dispose of lipofuscin by cellular division, a process that naturally results in dilution of lipofuscin [32]. Lipofuscin accumulation in aged tissues and age- related pathologies is considered a progressive phenomenon, probably as a consequence of the decline of the cellular clearance systems of misfolded (lipo)proteins and possibly other ‘aberrant’ metabolites [16, 33, 34]. We observed the lipofuscin granules in cells shortly after they became senescent. This fact implies that lipofuscin formation is possibly related to the senescent state, rather than just arandomtime-coincident event. Our findings reinforce the observation that senescent cells are hyper-metabolic and full of lipids in the cytoplasm. [35]. As no specific antibody for lipofuscin exists, we have used the SBB histochemical stain to demonstrate the presence of lipofuscin [16]. The dye is diluted in ethanol and due to its lipophilic nature, when in contact with lipids; it assembles on the lipid surface, as it is more soluble in lipids than in ethanol [26]. Accordingly, SBB stains the lipid component of lipofuscin [26]. Several methods were reported to detect lipofuscin, including fluorescence microscopy due to the natural auto-fluorescence of lipofuscin [36], as well as histochemical dyes like SBB, Berlin Blue, Nile Blue, Ziehl-Neelsen and Periodic acid Schiff [37]. As SBB is shown to specifically mask lipofuscin's fluorescence, it was considered the most reliable histochemichal stain to apply [19, 26, 27, 38]. SBB is suitable for use both in frozen and FFPE material [27]. We also verified that SBB staining for lipofuscin may depict senescent cells in FFPE sections from precancerous lesions already shown to contain senescent cells [5, 7, 28] (Fig. 7, 8A-C and Fig.9), while it is negative in the adjacent normal tissue (Fig. 8D) and the related carcinomas (Fig. 7) that have over-come the senescence anti-tumor barrier [5]. These characteristics support the candidacy of SBB for a highly desirable tool to study senescence in archival material.

There is a broadrange of potential senescence markers whose reliability, however,varies [39, 40]. None of the candidate markers proposed to date, however, is considered entirelyspecificfor cellular senescence, especially for *in vivo* applications[3]. The most commonly used senescence biomarker is SA-β-gal activity [14]. On the other hand, even the SA-β-gal assay apparently produces false positive results, under certain culture conditions such as confluence and serum starvation [29, 30>]. Also, cells that do not express the galactosidase gene, show no SA-β-gal activity, but fully execute the senescence program [31]. SA-β-gal, can only be used in fresh-frozen tissue as it is based on the enzymatic activity of galactosidase [14]. This limitation prevents to use SA-β-gal for studies of senescence in FFPE- archival material [1]. Furthermore, if cells or tissues are left in SA-β-gal solution for a prolonged time, all cells eventually will acquire the characteristic turquoise stain, due to their normal enzymatic galactosidase activity (Fig. 10C) [31]. To perform the technique a control sample is always required and the process is usually stopped when the sample under investigation starts to stain. As different tissues and cells require different times to stain and the desired stain is selected subjectively by the observer, the density and intensity of the turquoise color considered as positive varies between studies. On the other hand, SBB is a fast dye and it takes only few minutes to stain lipofuscin in tissues and cells, while the results are homogeneous and reproducible. This technique for identifying senescent cells is therefore easier, more rapid, likely more reproducible, and especially more suited for a wider spectrum of applications in diagnostic pathology laboratories. Moreover, SBB is a technique employable in frozen tissue [27], so it is also ideal for use in parallel with SA-β-gal as an additional biomarker of the senescent state. Of note, like all the other senescence biomarkers, lipofuscin is not 100% specific for senescence as it aggregates in degenerative conditions such as macular degeneration [16]. As shown (Fig. 10A, 10B) in confluent cultures, cells containing lipofuscin may be detected. However, in contrast to SA-β-gal activity which is immediately demonstrated in confluence and is present in all the cells (Fig. 10A), lipofuscin granules appear later and to a significantly lesser extent (Fig. 10B).

The majority of pathologies associated with lipofuscin are age-related diseases [16]. The role of cellular senescence in age-related pathologies and cancer, conditions that are both considered integral components of “aging”, is a widelygrowing fieldof biomedical research [39, 40]. Future studies will focus on the role of cellular senescence in cancer, especially considering the increasing evidence thatsenescence is one of major outcomes and a determinant of treatment response in oncology, broadly analogous to significance of apoptosis [13, 41]. Furthermore, there is evidence that presence of senescent cells in certain malignant tumors may be a sign of better prognosis [42]. There is little doubt that a convenient bio-marker applicable in archival tissue materialwould greatly facilitate research on cellularsenescence in cancer. Thedetectionoflipofuscin with SBB could be applied in studies that evaluate the effects of chemotherapy and otherantineoplastic treatments.

Taken together, our present study showed that lipofuscin can provide a senescence biomarker comparable to the SA-β-gal activity. Detection of lipofuscin with SBB stain can be applied as a stand-alone or auxiliary to SA-β-gal technique. Furthermore, SBB may visualize senescent cells in FFPE tissues, the most common form of archival clinical material, thereby extending the applicability of currently available candidate senescence biomarkers to a much wider selection of research topics related to diverse diseases and aging.

## METHODS

### Cells and inducible cellular systems

Primary human diploid lung fibroblast cultures (DLFs) were used as previously described [20]. Briefly, cells were grown in DMEM supplemented with 10% FCS (Gibco, AntiSel, Greece), 2 mmol/L L-Glutamine (Gibco, AntiSel, Greece), and 100 μg/mL penicillin and streptomycin (Gibco, AntiSel, Greece), respectively, at 37°C and 5% CO2. The cells were serially sub-cultured at 1:2 split ratio until replicative senescence (42 passages). Alternatively, early passage fibroblasts (7 passages) were repeatedly exposed, twice a day, to sub-lethal doses (4 Gy) of γ-radiation up to a cumulative dose of approx. 50 Gy (12×4Gy), in a ^60^Co gamma source (Gamma Chamber 4000A, Isotope Group, Bhadha Atomic Research Company, Trombay, Bombay, India) at a rate of 8 Gy/min. The cells were subcultured and after additional two weeks, fixed and analyzed. The regulatable osteosarcoma cell models Saos-2 p21 Tet-On, Saos-2 p53 Tet-On and U2OS-E2F1-ER were cultured as previously described [25, 43]. Briefly, these cell lines were maintained under the same culture conditions as DLFs, except for the use of FBS tetracycline-free medium (Clontech, Lab Supplies, Greece) on Saos2-p21 Tet-On and Saos2-p53 Tet-On systems. The U2OS-E2F1-ER system induction was accomplished with 300nM 4-OH-Tamoxifen, for 10 days. Saos-2 p21 Tet-On and Saos-2 p53 Tet-On systems were induced with 5μg/ml doxycyclin for 8 days before fixation. All cell lines were fixed in 4% parafolmadehyde (5 min, room temperature) then washed with sterile PBS and kept at 4°C until staining.

### Human samples and animal models

Tissue samples from lung adenomas and adenocarcinomas from a mouse model expressing conditionally K-*rasV12*in the lung were analyzed [5]. Normal mouse lung tissue was obtained from the Laboratory of Cell Proliferation and Ageing, Institute of Biology, National Centre for Scientic Research ‘Demokritos. Subsequently, surgically removed material from patients with benign prostate hyperplasia was obtained (with consent of patients according to the National Kapodistrian University of Athens ethical committee guidelines). From each sample, material was partitioned and either stored at −80°C [14] or routinely formalin-fixed and paraffin-embedded (FFPE).

### SA-β-galactosidase Assay

The activity of SA-β-gal in cell cultures and frozen tissues was detected according to Debacq-Chainiaux *et al.*[14]. Cells with cytoplasmic staining were scored as positive. Fresh-frozen, formaldehyde-fixed SA-β-gal pre-incubated tissues were stained as described elsewhere[6].

### Lipofuscin staining protocol: Sudan Black B (SBB) staining

Combining the protocols of Gatenby *et al*. [17], and Rasmussen, [18] for SBB staining, we achieved optimal lipofuscin visualization in cell cultures and tissues with the following methodology:

*Preparation of SBB solution:* 0.7gr of SBB (BDH, Vizas, Athens, Greece) was dissolved in 70% ethanol, covered with parafilm and thoroughly stirred overnight at room temperature. Filtered through filter paper and then filtered again through frittered glass filter of medium porosity with suction. Throughout the process, it was important to avoid ethanol evaporation,which results in precipitation of the stain, so the solution was storedin an airtightcontainer.
*Staining Procedure:* OCT-Frozen-sections mounted onto superfrost slides were fixed in 1% (wt/vol) formaldehyde/PBS for 1 min at room temperature and then washed three times (approx.1 min) at room temperature, with PBS. Sections were then incubated for 5 min in 50% ethanol and then for another 5 min into 70% ethanol. Coverslips with fixed cells were incubated for 2 min in 70% ethanol. Tissue samples were dewaxed with xylene and dehydrated until 70% ethanol. In order to avoid precipitation of SBB on cells or tissues the following two steps are crucial: 1) a drop from freshly prepared SBB was dropped on a clean slide. The coverslip with the cells or the dehydrated tissue on a slide was placed facing down on the drop of SBB on the slide. The staining was observed under the microscope. The desirable outcome with no precipitation was accomplished by 2-8 minutes. 2) The coverslip or the slide, were carefully lifted and the SBB on the edges of the coverslip or the tissue-slide was wiped out manually from the back and along the edges of the coverslip or the slide with the help of a soft paper. The cells or the tissues were then embedded into 50% ethanol, transferred and washed in distilled water, counterstained with 0.1% Nuclear Fast Red (NFR) (Sigma, BioLine, Athens Greece) for 10 min., and mounted into 40% Glycerol/TBS mounting medium. Lipofuscin staining was considered positive when perinuclear and cytoplasmic aggregates of blue-black granules were evident inside the cells.

### Immunofluorescence

For indirect immunofluorescence analysis, cells were fixed with 4% paraformaldehyde in PBS and subsequently incubated with the primary antibody anti-E2F1 (1:100) (KH-95, Santa Cruz, Bioanalytica, Athens, Greece), as previously described [25].

### Immunoblotting analysis

Total protein extraction from cells and SDS-polyacrylamide gel electrophoresis was performed as previously described [25]. The antibodies used were: anti-p21 (1:500) (F-5, Santa Cruz, Bioanalytica, Athens, Greece) and anti-p53 (1:500) (DO-1, Santa Cruz, Bioanalytica, Athens, Greece).

### Auto-fluorescence Detection of Lipofuscin

The FFPE tissue sections were deparaffinized, hydrated and mounted into 40% glycerol/TBS mounting medium. The cells after fixation were also mounted into the same mounting medium. Lipofuscin auto-fluorescence was then evidenced by excitation at 450-490nm, using a dichromatic mirror at 510nm and a long-pass filter at 515nm [26]. We used the Leica DMRAZ microscope equipped with the Leica DFC350FX camera. This analysis was performed in cultured young and senescent cells as well as in all tissue sections. Lipofuscin auto-fluorescence was quenched with 0.7% SBB in 70% ethanol [38].
